# Mediterranean fever gene variants may prevent the development of lupus nephritis in Japanese patients with systemic lupus erythematosus

**DOI:** 10.3389/fimmu.2025.1571208

**Published:** 2025-07-07

**Authors:** Yushiro Endo, Tomohiro Koga, Lamiaa Mohamed, Yoshika Tsuji, Masataka Umeda, Hiroko Hayashi, Tatsuya Kishino, Atsushi Kawakami

**Affiliations:** ^1^ Department of Immunology and Rheumatology, Division of Advanced Preventive Medical Sciences, Nagasaki University Graduate School of Biomedical Sciences, Nagasaki, Japan; ^2^ Department of Pathology, Sasebo City General Hospital, Sasebo, Japan; ^3^ Division of Functional Genomics, Nagasaki University Graduate School of Medical Sciences, Nagasaki, Japan

**Keywords:** systemic lupus erythematosus, Mediterranean fever variant, lupus nephritis, adaptive immunity, innate immunity

## Abstract

**Background:**

Systemic lupus erythematosus (SLE) is an autoimmune disease characterized by loss of immune tolerance, leading to systemic inflammation and organ damage. The *Mediterranean fever* (*MEFV*) gene, primarily linked to familial Mediterranean fever (FMF), has been suggested to have a protective role against SLE. However, comprehensive whole-exon analyses of *MEFV* and research on *MEFV* or FMF in non-Mediterranean populations, where *MEFV* exon 10 mutations are relatively rare, are limited.

**Methods:**

We conducted a whole-exon analysis of the *MEFV* gene in 55 Japanese patients with SLE. Patients were classified based on the presence or absence of *MEFV* variants, and their clinical characteristics were compared. In addition, we generated MRL/lpr mice with the human *MEFV* E148Q variant using CRISPR technology to examine its impact on disease phenotypes. Disease activity and kidney pathology were assessed using the established clinical and histological scoring systems.

**Results:**

Among the 55 patients, those carrying *MEFV* variants exhibited a significantly lower prevalence of lupus nephritis than non-carriers (P = 0.007). The number of *MEFV* variants was inversely associated with the risk of lupus nephritis (P = 0.03). In MRL/lpr mice, the E148Q variant was associated with reduced anti-dsDNA antibody production, reduced formation of memory B cells, and milder kidney pathology, indicating a shift from adaptive immunity to innate immune responses.

**Conclusions:**

Our findings suggest that *MEFV* variants, particularly the E148Q variant, may play a protective role against lupus nephritis in Japanese patients with SLE by modulating immune responses. These results provide valuable insights into the genetic factors influencing SLE severity.

## 1 Introduction

Systemic lupus erythematosus (SLE) is a heterogeneous disease characterized by loss of immune tolerance and autoantibody production against elements of the cell nucleus followed by immune-mediated tissue damage and systemic inflammation (1, 2). More than 100 susceptibility loci have been identified through genome-wide association studies as potential genetic factors contributing to the development of SLE (3–9).

The prevalence of *Mediterranean fever* (*MEFV*) gene variants is lower in adult patients with SLE than in healthy individuals (10). The prevalence of SLE is also lower in several large cohorts of patients with familial Mediterranean fever (FMF) compared with healthy individuals (11–14). These results suggest a potential protective effect of *MEFV* variants and/or FMF against SLE. *MEFV* variants may modify clinical SLE phenotypes (10, 15, 16). In particular, the E148Q variant in the *MEFV* exon 2 may prevent the development of lupus nephritis in adult patients with SLE (15). However, reports showing a relationship between SLE and *MEFV* gene variants/mutations are limited to patients in the Mediterranean area, where *MEFV* exon 10 mutations are relatively common. In addition, whole-exon analyses to determine the relationship between *MEFV* gene variants and SLE have not been reported.

A relatively high proportion of healthy individuals in East Asian population including Japan have *MEFV* exon 2 and exon 3 variants (especially E148Q variants in *MEFV* exon 2) as follows: minor allele frequency (MAF) for E148Q, 1443 out of 5160 alleles (28.0%) in East Asian population and 3555 out of 147018 (2.4%) in non-East Asian population based on a genome database from gnomAD v3.1.2 (https://gnomad.broadinstitute.org). The genetic characteristics of the *MEFV* gene in Japan and the Mediterranean region are different. We performed a whole-exon analysis of the *MEFV* gene in 55 Japanese patients with SLE and compared the clinical characteristics of SLE in patients with and without *MEFV* variants. MRL/lpr mice with the human *MEFV* E148Q variant were generated to confirm the contribution of *MEFV* variants with the E148Q to SLE clinical phenotypes.

## 2 Methods

### 2.1 Study design

This study included 55 Japanese patients with SLE treated between January 2008 and April 2020 at Nagasaki University Hospital. All patients met the American College of Rheumatology 1997 revised criteria (17) and/or the Systemic Lupus International Collaborating Clinics 2012 criteria (18) for SLE classification and were diagnosed with SLE based on their clinical courses. Patients were clinically observed for ≥12 months. This study was approved by the Institutional Review Board of Nagasaki University (approval no. 18070919), and all patients provided signed informed consent to participate in the study.

### 2.2 Genetic analysis

Genomic DNA was extracted from whole blood using the Promega Wizard^®^ Genomic DNA Purification Kit (Promega, Madison, WI, USA). Whole-exon analyses was performed to detect single nucleotide variants and short insertions and deletions in *MEFV* gene via targeted next-generation sequencing. Next-generation sequencing (NGS) was performed on an Ion Personal Genome Machine (Thermo Fisher Scientific, Waltham, MA, USA) using an Ion AmpliSeq Library Kit Plus (Thermo Fisher Scientific). Each depth of coverage of the variants detected by NGS was analyzed to show the quality. The variant allele frequencies in the patients were almost 50% or almost 100%. No chimeras or mosaics were identified. The number of *MEFV* variants for each patient was counted; heterozygous variants were counted as one and homozygous variants were counted as two.

### 2.3 Clinical and laboratory assessments

Demographic data, laboratory results, and disease activity at the time of SLE diagnosis were obtained from patient records. Laboratory assays were performed in a routine clinical setting. Disease activity was objectively measured at the time of SLE diagnosis using the Safety of Estrogens in Lupus Erythematosus National Assessment–Systemic Lupus Erythematosus Disease Activity Index (SELENA-SLEDAI) score. The score range for the SELENA-SLEDAI is 0–105, and higher scores indicate increased disease activity (19).

Clinical characteristics and comorbidities were assessed during the clinical course of the disease. Lupus nephritis was diagnosed based on pathological findings from renal biopsies and/or the clinical course. Renal pathologists classified renal biopsies according to the International Society of Nephrology/Renal Pathology Society guidelines (20).

### 2.4 Generation of human *MEFV exon 2* encoding wild-type and E148Q knock-in MRL/lpr mice

Wild-type MRL*.lpr* mice were obtained from Jackson Laboratory and maintained under specific pathogen-free conditions at the animal facility of Nagasaki University. The animal study protocol was approved by the Institutional Animal Care and Use Committee of Nagasaki University.

The following guide RNA targeting exon 2 of mouse *MEFV* was designed using CRISPR Design Tools (https://horizondiscovery.com/en/ordering-and-calculation-tools/crispr-design-tool): 5′-CCATCCAGCCAGGCTGAAGTGTTTTAGAGCTATGCT-3′. The following single-strand DNA oligonucleotides (ssODN) were used to introduce the human MEFV exon 2 sequence into the mouse counterpart: Wild-type human *MEFV* exon 2: 5′-CCTGAAGGTGATGGGACACAGCAAAACAATGATGAATCAGACACCCTTCCATCCAGCCAGCCC**GAG**GCCGGGAGGGGGCTGTCGAGGAAGTCACTGACCAAAAGGAAGGATCAGAGGGGCCCCGAGAGCCTGGACTCACAGACCAAG-3′, human *MEFV* E148Q variant: 5′-CCTGAAGGTGATGGGACACAGCAAAACAATGATGAATCAGACACCCTTCCATCCAGCCAGCCC**CAG**GCCGGGAGGGGGCTGTCGAGGAAGTCACTGACCAAAAGGAAGGATCAGAGGGGCCCCGAGAGCCTGGACTCACAGACCAAG-3′. A mixture of CAS9 nuclease mRNA (10 ng/μL), crRNA (100 ng/μL), tracrRNA (200 ng/μL), and ssODN (10 μM) was injected into the cytoplasm of MRL/lpr zygotes using an Olympus IX70 Fluorescence Microscope (Olympus, Tokyo, Japan), TransferMan NK2 (Eppendorf, Hamburg, Germany), FemtoJet 4i (Eppendorf), and CellTram 4r Oil (Eppendorf). CAS9 nuclease mRNA was purchased from GE Healthcare Dharmacon (Lafayette, CO, USA), and other reagents were purchased from Integrated DNA Technologies (Coralville, IA, USA). Zygotes were cultured until the two-cell stage *in vitro* before transferring to recipient pseudopregnant female oviducts. MRL/lpr exhibiting the homozygous wild-type human *MEFV* exon 2 (MRL/lpr^hMEFV WT^) and the homozygous human *MEFV* E148Q variant (MRL/lpr^hMEFV E148Q^) were used for comparisons.

### 2.5 Proteinuria in MRL/lpr mice

Urine was collected at 8, 10, and 16 weeks from each female MRL/lpr^hMEFV WT^ and MRL/lpr^hMEFV E148Q^ mice (n = 3 per group). Urinary creatinine (urinary) was measured with a colorimetric assay kit (Cayman Chemical) and mouse albumin was measured with an ELISA kit (Bethyl Laboratories). Proteinuria was calculated as the albumin-to-creatinine ratio.

### 2.6 Serum autoantibody in MRL/lpr mice

Serum samples were collected at 8 and 16 weeks from female mice in each group (n = 3 per group). Serum anti-dsDNA IgG levels were measured using a mouse anti-dsDNA IgG ELISA kit (Alpha Diagnostic Intl. Inc.) according to the manufacturer’s instructions.

### 2.7 *In vitro* bone marrow-derived macrophage differentiation

Bone marrow-derived macrophages (BMDMs) were obtained by differentiating bone marrow progenitors from the tibia and femur of 8-week-old female mice for 7 days in Iscove’s Modified Dulbecco’s Medium containing 20 ng/ml M-CSF (Peprotech), 10% heat-inactivated fetal bovine serum (Invitrogen), 1 mM sodium pyruvate, 100 U/ml penicillin, and 100 Mg/ml streptomycin (Invitrogen). BMDMs were plated in 12-well plates 24 h before experiments and stimulated with LPS (1,000 ng/ml) for 5 h. The supernatants of culture media were stored at −80°C until the following assessment. Cytokines were measured in the supernatants from each group (n = 3 per group) using a cytokine bead assay and magnetic beads (Millipore, Billerica, MA, USA), according to the manufacturer’s instructions.

### 2.9 Flow cytometry

Spleens were excised from 16-week-old female mice (n = 5 per group), and cell suspensions were obtained by teasing the organs through a nylon mesh. Isolated cells were stained for 30 min at 4°C with the following antibodies: anti-mouse CD3 (clone 17A2, BioLegend), anti-mouse CD19 (clone 6D5, BioLegend), and anti-mouse IgD (clone 11-26c.2a, BioLegend). Data were acquired using an LSRII flow cytometer (BD Biosciences) and analyzed using FlowJo v. 7.6.1.

### 2.10 Assessment of skin and kidney pathology

The skin and kidneys were collected from 16-week-old female mice in each group (n = 5–6 per group). Tissues were stored in 4% paraformaldehyde, sectioned, stained with hematoxylin and eosin (H&E), and scored by a certified pathologist. The skin severality score was calculated as the sum of cellular infiltration (score 0–3), epidermal hyperplasia (score 0–2), and epidermal ulcerations (score 0–2) based on a previously described method (21). The kidney severality scores were determined using a previously described method (https://www.inotiv.com/solutions/mouse-lupus-methods). Subsequently, glomerular scores, focusing on the glomerular area including the crescent, were also assessed according to previously described criteria (22). Briefly, twenty glomeruli were randomly observed per mouse using H&E-stained slides. Each glomerulus was scored using a semi-quantitative scale, as follows: 0 normal (35–40 cells/gcs), 1 mild (glomeruli with slight proliferative changes and mild hypercellularity, 41–50 cells/gcs, and/or minor exudation), 2 moderate (glomeruli with moderate hypercellularity, 50–60 cells/gcs, including segmental and/or diffuse proliferative changes, hyalinosis, and/or moderate exudates), and 3 severe (glomeruli with segmental or global sclerosis and/or exhibiting severe hypercellularity, 60 cells/gcs, crescent formation, and/or heavy exudation).

### 2.11 Statistical analysis

Categorical variables are presented as frequencies and quantitative variables are presented as medians and interquartile ranges. Categorical variables were compared using Fisher’s exact tests, and quantitative variables were compared using Wilcoxon rank sum tests. Multiple logistic regression analysis was performed to identify independent factors associated with lupus nephritis as the primary endpoint. Clinically important variables were included in the model. Mouse groups were compared using unpaired Student’s *t*-tests after the assumption of normality was tested for the datasets using the Shapiro-Wilk test. All statistical analyses were performed using JMP Pro 18.1.1 software (SAS Institute, Cary, NC, USA). P < 0.05 (two-tailed) was considered statistically significant. GraphPad Prism version 8.0 was used to create figures. As this was an exploratory study aimed at identifying potential associations and generating hypotheses, no corrections for multiple comparisons were applied. Results should therefore be interpreted with caution, and future confirmatory studies are warranted to validate the findings.

## 3 Results

### 3.1 Characteristics and *MEFV* variants in patients with SLE

The demographic and clinical characteristics and mutational pattern of the *MEFV* gene in patients with SLE are summarized in **Supplementary Table 1**. All patients with SLE and *MEFV* variants had one or more of the following variants in exons 1–5 of *MEFV*: E84K, L110P, E148Q, R202Q, G304R, P369S, R408Q, and S503C. None of the patients had exon 10 mutations. The depth of coverage of all *MEFV* variants detected by NGS was 736 ± 74.45 (Average ± SEM).

### 3.2 Characteristics of SLE patients with *MEFV* variants

The demographic and clinical characteristics of patients with SLE without *MEFV* variants (*MEFV* variant non-carriers, n = 16) and patients with SLE and *MEFV* variants (*MEFV* variant carriers, n = 39) were compared (**Table 1**). *MEFV* variant carriers exhibited a significantly lower prevalence of lupus nephritis compared with non-carriers (*P* = 0.007). No significant differences in other parameters were detected between carriers and non-carriers. The demographic and clinical characteristics of SLE in patients with (n = 22) or without (n = 33) the E148Q variant in exon 2 of *MEFV* were also compared. No significant difference in the prevalence of lupus nephritis was detected between *MEFV* E148Q carriers and non-carriers (39.4% vs. 54.6%, *P* = 0.29, not shown).

**Table 1:** Clinical characteristics of patients in the presence or absence of *MEFV* variants.

Variables	*MEFV* variant non-carriers (n = 16)	*MEFV* variant carriers (n = 39)	P-value
Patient characteristics			
Age at onset (years) *	30.5 (17.8–45.8, 16)	30.0 (21.0–39.0, 39)	0.85
Later-onset SLE (%)	2/16 (12.5%)	4/39 (10.3%)	1.00
Male gender (%)	4/16 (25.0%)	3/39 (7.7%)	0.18
Family history of autoimmune disease (%)	6/16 (37.5%)	9/39 (23.1%)	0.33
Family history of autoinflammatory disease (%)	0/16 (0.0%)	1/39 (2.6%)	1.00
Clinical characteristics and comorbidities			
The other autoimmune disease (%)	5/15 (33.3%)	9/36 (25.0%)	0.73
High fever (%)	7/15 (13.3%)	17/36 (47.2%)	1.00
Headache (%)	4/15 (26.7%)	12/36 (33.3%)	
Pleurisy (%)	3/15 (20.0%)	9/36 (25.0%)	1.00
Peritonitis (%)	0/15 (0.0%)	1/36 (2.8%)	1.00
Pericarditis (%)	0/15 (0.0%)	7/36 (19.4%)	0.09
Arthritis (%)	11/15 (73.3%)	24/36 (66.7%)	0.75
Skin involvement (%)	10/15 (66.7%)	22/36 (61.1%)	0.76
Neuropsychiatric involvement (%)	4/15 (26.7%)	9/36 (25.0%)	1.00
Lung involvement (%)	1/15 (6.7%)	4/36 (11.1%)	1.00
Lupus nephritis (%)	12/16 (75.0%)	13/39 (33.3%)	0.007
Classification of biopsy-proven lupus nephritis	Class II, 3; Class III, 1; Class IV, 1; Class V, 3	Class II, 2; Class IV, 5; Class V, 2; Class III + V, 1; Class IV + V, 3	NA
SELENA-SLEDAI score at disease onset *	15.0 (8.5–19.0, 13)	11.0 (8.0–21.5, 34)	0.83
Laboratory findings at disease onset			
WBC (x10^3^/μl) *	4.2 (2.4–5.2, 12)	4.1 (2.9–5.3, 31)	0.69
Hemoglobin (g/dl) *	11.9 (10.5–12.2, 12)	11.0 (9.9–12.2, 31)	0.30
PLT (x10^4/^ul) *	19.5 (12.7–24.8, 12)	18.1 (9.4–26.0, 31)	0.99
CRP (mg/dl) *	0.4 (0.1–1.3, 12)	0.2 (0.04–0.5, 31)	0.10
Low C3 (%)	7/12 (58.3%)	20/31 (64.5%)	0.74
Low C4 (%)	6/12 (50.0%)	16/31 (51.6%)	1.00
Low CH50 (%)	6/12 (50.0%)	20/31 (64.5%)	0.49
Anti-dsDNA antibody positivity (%)	11/15 (73.3%)	25/35 (71.4%)	1.00

*Median (interquartile range, number) or number (percentages) are shown. P-values were established using Fisher’s exact test or the Mann-Whitney U-test. SLE, systemic lupus erythematosus; MEFV, Mediterranean Fever; SELENA-SLEDAI, Safety of Estrogens in Lupus Erythematosus National Assessment–Systemic Lupus Erythematosus Disease Activity Index; WBC, white blood cell count; PLT, Platelet; CRP, C-reactive protein; NA, Not available.

### 3.3 Number of *MEFV* variants according to the presence of lupus nephritis

The number of *MEFV* variants in patients with lupus nephritis was significantly lower than the number of *MEFV* variants in patients without lupus nephritis (Mean ± SD; 0.80 ± 0.87 vs. 1.53 ± 0.94, = 0.006, not shown). The number of *MEFV* variants, but not low CH50 or anti-dsDNA antibody positivity, was significantly associated with lupus nephritis in patients with SLE (odds ratio 0.351, P = 0.03; **Table 2**).

**Table 2:** Multiple regression analysis of factors associated with the development of lupus nephritis in patients with systemic lupus erythematosus.

Endpoint	Variables	Definition / Unit	Odds rate (95% CI)	P-value
Lupus nephritis	Low CH50	Presence	4.610 (0.913–29.243)	0.08
	Anti-dsDNA antibody positivity	Presence	4.544 (0.825–36.551)	0.10
	Total number of *MEFV* variants	1 increase	0.351 (0.122–0.797)	0.03

The odds ratio, 95% confidence interval (95% CI), and p-value of each value for the endpoint, lupus nephritis, are shown. SLE, systemic lupus erythematosus; MEFV, Mediterranean Fever.

### 3.4 MRL/lpr mice with human exon 2 of the *MEFV* gene

**Figure 1:**
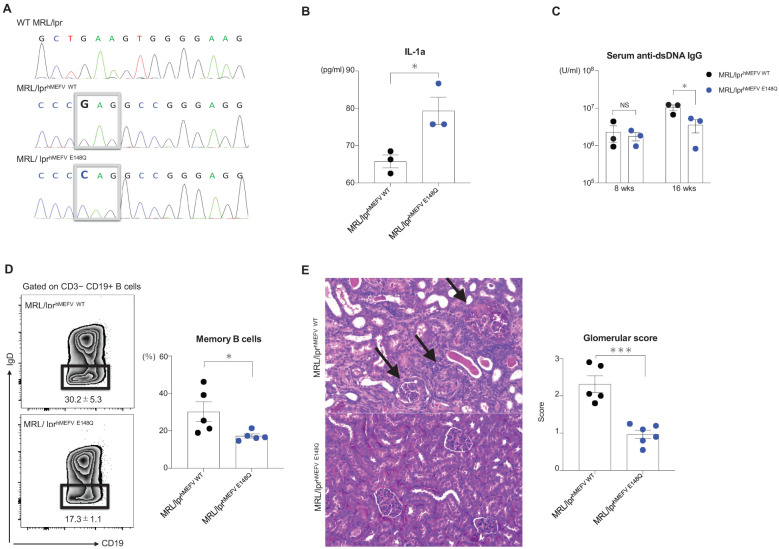
MRL/lpr mice with human exon 2 of the *MEFV* gene. MRL/lpr^hMEFV WT^ and MRL/lpr^hMEFV E148Q^ mice were compared. **(A)** Sanger sequencing results and parts of *MEFV* exon 2 in wild-type MRL/lpr, MRL/lpr^hMEFV WT^, and MRL/lpr^hMEFV E148Q^. The boxed sequences represent glutamic acid in MRL/lpr^hMEFV WT^ and glutamine in MRL/lpr^hMEFV E148Q^. **(B)** Supernatant IL-1α levels from cultured BMDMs isolated from MRL/lpr^hMEFV WT^ and MRL/lpr^hMEFV E148Q^ mice. **(C)** Anti-dsDNA IgG levels at 8 and 16 weeks. **(D)** Representative flow cytometric plot of splenic class-switched memory B cells (CD3−CD19+IgD+) and the percentage of splenic memory B cells in each group. **(E)** Representative H&E-stained kidney sections and glomerular severity scores in each group. The black arrows show crescent formations in the glomerulus. **(B-E)** Unpaired two-tailed Student’s t-test. Data represent means ± SEMs (NS; not significant, *P < 0.05, ***P < 0.005.

Successful insertion of the human *MEFV* exon 2 sequence into the mouse counterpart was confirmed via Sanger sequencing (**Figure 1A**). IL-1α levels were significantly higher in the media of BMDMs from MRL/lpr^hMEFVE148Q^ mice compared with the levels in the supernatants of BMDMs from MRL/lpr^hMEFV WT^ mice (**Figure 1B**). In contrast, anti-dsDNA antibody production (**Figure 1C**) and memory B cell formation in spleens were reduced in MRL/lpr^hMEFVE148Q^ mice compared with MRL/lpr^hMEFV WT^ mice (**Figure 1D**). No significant differences in the skin severity scores and proteinuria levels were detected between the groups (**Supplementary Figures S1A, B**). However, kidney severity scores in MRL/lpr^hMEFV E148Q^ mice were lower than kidney scores from MRL/lpr^hMEFV WT^ mice, particularly in the glomerular region, including reduced crescent formation (**Supplementary Figure S1C**). Glomerular severity scores were also significantly lower in the MRL/lpr^hMEFV E148Q^ mice (**Figure 1E**).

## 4 Discussion

Our results provide compelling evidence for the protective role of *MEFV* variants, including the E148Q variant, in shaping the clinical trajectory of SLE; *MEFV* variants may mitigate the severity of lupus nephritis. Clinical observations in Japanese patients with SLE combined with experimental results from a murine model highlight the contribution of the E148Q variant in redirecting immune responses from adaptive immunity toward innate immune mechanisms.

The role of *MEFV* variants (excluding homozygous exon10 mutations but including the E148Q variant) in the development of autoinflammation is controversial. However, previous reports suggest that *MEFV* variants contribute to the pathogenesis of other autoinflammatory diseases (23–25). Our clinical findings demonstrate a significantly lower prevalence of lupus nephritis in SLE patients carrying *MEFV* variants compared with non-carriers. Of note, the number of *MEFV* variants inversely associated with the risk of lupus nephritis in patients with SLE, underscoring the protective effects of *MEFV* variants. These results align with a previous study suggesting that the E148Q variant prevents lupus nephritis (15). These results suggest a shift in immune responses from autoimmunity in SLE to autoinflammation. Notably, the protective effects of *MEFV* variants in this cohort were not solely due to the E148Q variant, emphasizing the need for larger-scale investigations using comprehensive whole-exon analyses of the *MEFV* gene in patients with SLE, similar to our approach.

Based on the kidney pathology in lupus-prone MRL/lpr mice, the severity of lupus nephritis was reduced in mice carrying the human *MEFV* E148Q variant. Notably, anti-dsDNA antibody production and memory B cell formation in the spleen were reduced in MRL/lpr^hMEFV E148Q^ mice, indicating suppression of adaptive immune responses. Conversely, IL-1α production by bone marrow-derived macrophages (BMDMs) from these mice increased compared with MRL/lpr^hMEFV WT^ mice, indicating that the variant enhanced the innate immune response. These results support the hypothesis that the E148Q variant promotes a shift from pathogenic adaptive immunity in SLE to innate immune activation, which may mitigate autoimmune-driven kidney damage. This shift from adaptive to innate immunity, mediated by *MEFV* variants such as E148Q, may reduce the severity of lupus nephritis by reducing autoantibody production.

This study provides valuable insights, but several limitations should be acknowledged. First, the size of the clinical cohort was small, limiting the generalizability of our results. No MEFV exon 10 mutations were detected among the patients enrolled in this study. While this finding may raise the hypothesis that MEFV exon 10 mutations could be protective against SLE, such an association is difficult to assess in the Japanese population due to the extremely low prevalence of these mutations. For example, the M694I mutation—the most frequently reported MEFV exon 10 variant in Japan—has a minor allele frequency of approximately 0.15% according to the Tohoku Medical Megabank Organization database (https://jmorp.megabank.tohoku.ac.jp). Given this rarity, it is not feasible to perform robust statistical analyses, such as Hardy–Weinberg equilibrium testing, in our cohort. Therefore, we believe that it is difficult to draw reliable conclusions regarding the association between exon 10 mutations and SLE susceptibility in this population. Larger, multicenter studies are necessary to confirm the protective role of *MEFV* variants and mutations in SLE among diverse populations.

Second, the experimental mouse model was informative but may not fully replicate the complexity and heterogeneity of patients with SLE. Finally, the precise molecular pathways mediating the protective effects of the E148Q variant against nephritis are unclear. The future investigations may provide insights into novel therapeutic strategies for SLE.

Finally, this study was exploratory in nature and intended to generate hypotheses regarding the potential involvement of MEFV variants in SLE pathogenesis. Given this objective, we did not apply corrections for multiple testing, as such adjustments may increase the risk of type II errors and obscure meaningful associations, especially when analyzing rare variants. Nonetheless, we acknowledge that the lack of multiple testing correction may increase the risk of false-positive findings. This limitation has been clearly stated, and our findings should be interpreted with appropriate caution. Future studies with larger cohorts and confirmatory designs are needed to validate these results.

In summary, the E148Q variant of the *MEFV* gene may protect patients with lupus nephritis by modulating immune responses and shifting the balance from adaptive to innate immunity. These findings highlight the protective role of *MEFV* variants and provide the foundation for further research on the innate immune mechanisms underlying SLE.

## Data Availability

The original contributions presented in the study are included in the article/**Supplementary Material**, further inquiries can be directed to the corresponding author/s.

## Supplementary material

Supplementary Figure 1 Comparison of MRL/lpr^hMEFV WT^ and MRL/lpr^hMEFV E148Q^ mice. **(A)** Skin severity scores based on hematoxylin and eosin (H&E)-stained skin sections. **(B)** Proteinuria levels are indicated by albumin-to-creatinine ratios at 8, 10, and 16 weeks. **(C)** Kidney severity scores based on H&E-stained kidney sections. Unpaired two-tailed Student’s t-test. Data and bars represent means ± SEM (NS; Not significant).
