# Supplementary figures and images for: Mediterranean fever gene variants may prevent the development of lupus nephritis in Japanese patients with systemic lupus erythematosus
Source: Front Immunol. 2025 Jul 7;16:1571208. doi: 10.3389/fimmu.2025.1571208 (PMC12278986; doi:10.3389/fimmu.2025.1571208)

A

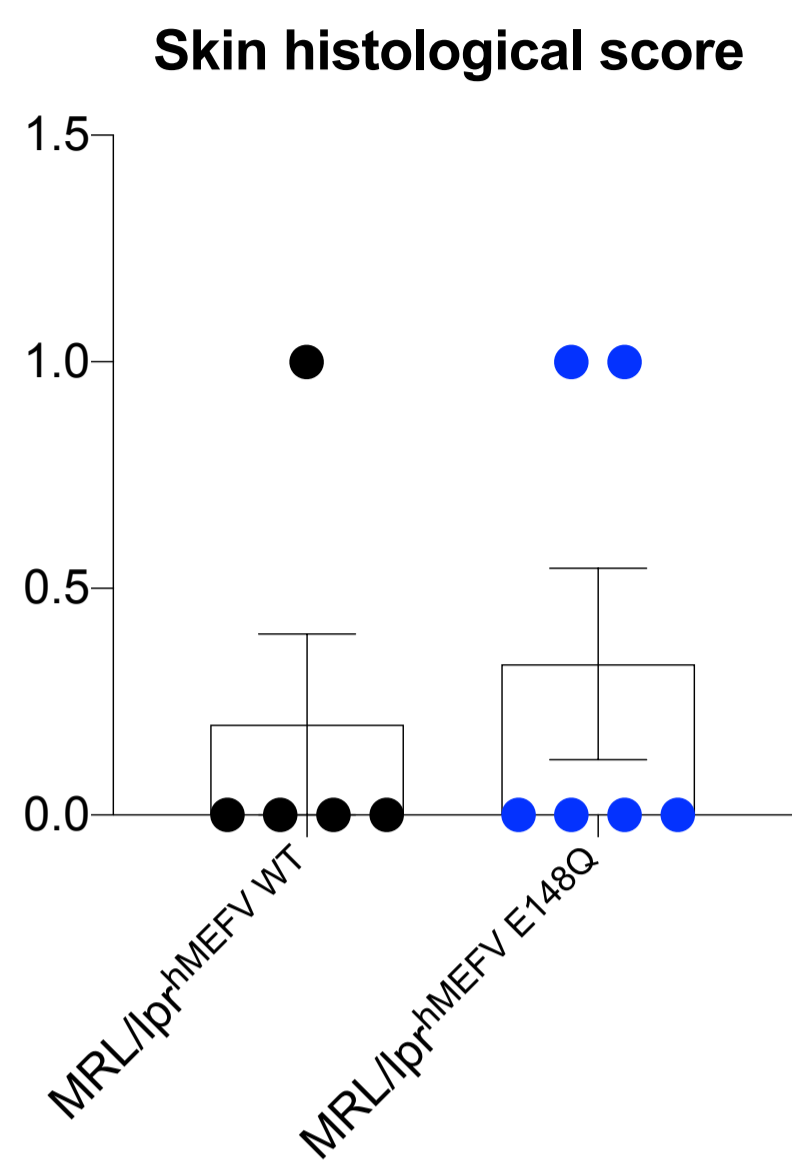

B

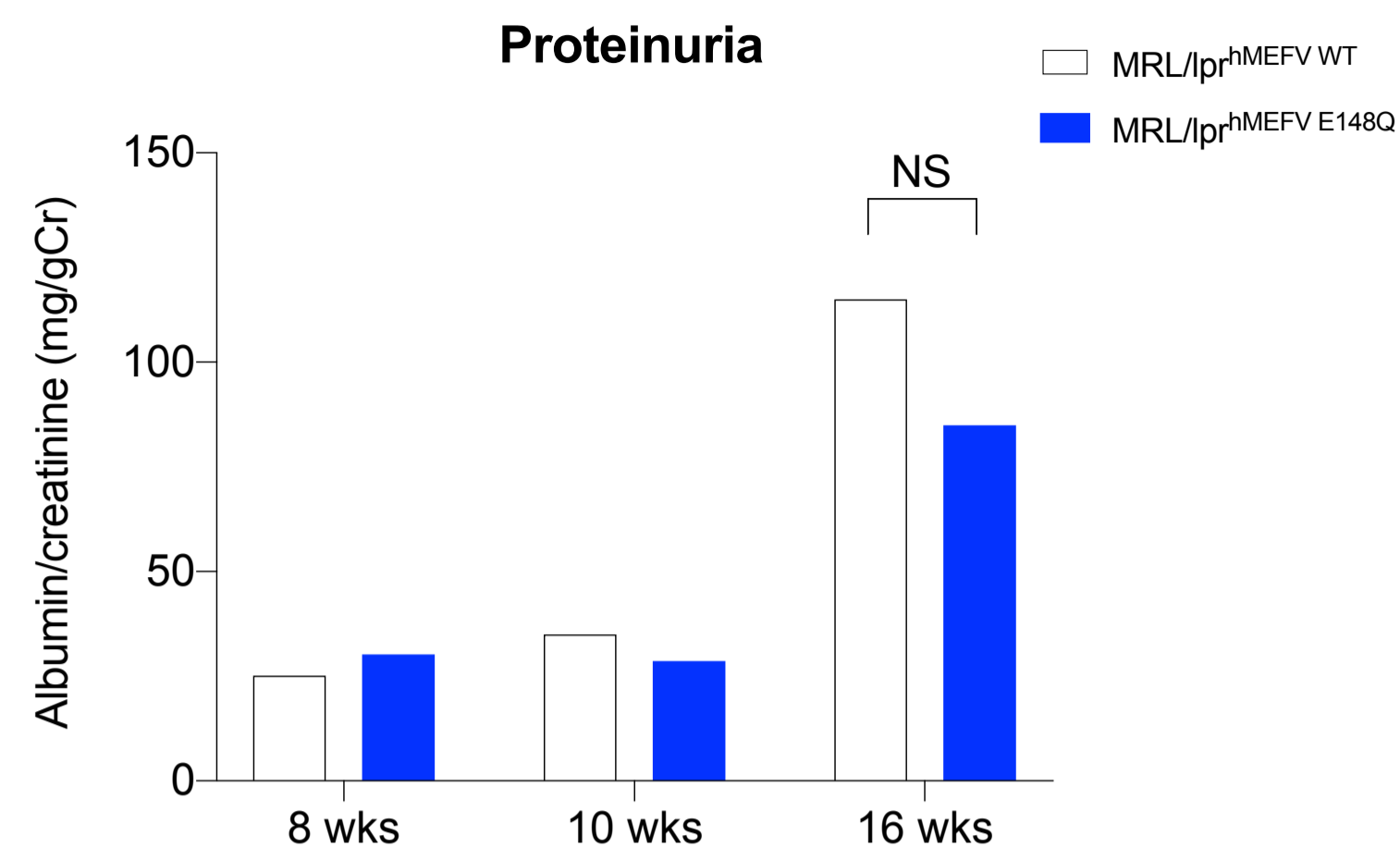

C

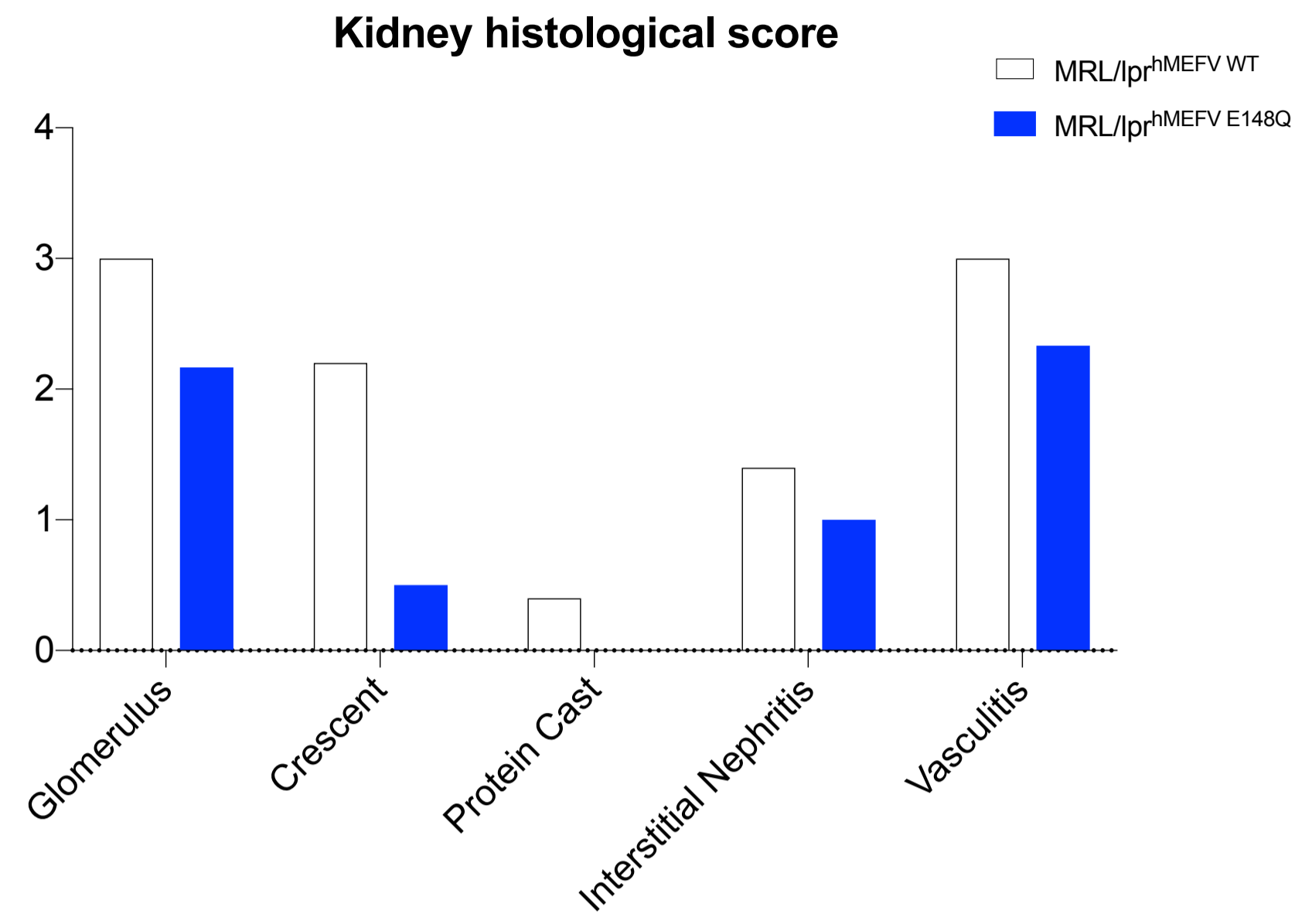

Supplement: Supplementary Figure 1 — Comparison of MRL/lprhMEFV WT and MRL/lprhMEFV E148Q mice. (A) Skin severity scores based on hematoxylin and eosin (H&E)-stained skin sections. (B) Proteinuria levels are indicated by albumin-to-creatinine ratios at 8, 10, and 16 weeks. (C) Kidney severity scores based on H&E-stained kidney sections. Unpaired two-tailed Student’s t-test. Data and bars represent means ± SEM (NS; Not significant). [file Image1.pdf]
