# Supplementary material for: Mediterranean fever gene variants may prevent the development of lupus nephritis in Japanese patients with systemic lupus erythematosus
Source: Front Immunol. 2025 Jul 7;16:1571208. doi: 10.3389/fimmu.2025.1571208 (PMC12278986; doi:10.3389/fimmu.2025.1571208)
Supplement: Supplementary file 2 [file Table1.pdf]

Supplemental Table 1. Demographic and clinical characteristics and *MEFV* variants in patients with SLE

Variables	All patients (n = 55)	Healthy controls in East Asian population #
Patient characteristics		
Age at onset (years) *	30.0 (21.0–41.0, 55)	NA
Later-onset SLE (%)	6/55 (10.9%)	NA
Male gender (%)	7/55 (12.7%)	NA
Family history of autoimmune disease (%)	15/55 (27.3%)	NA
Family history of autoinflammatory disease (%)	1/55 (1.8%)	NA
Clinical characteristics and comorbidities		
The other autoimmune disease (%)	14/51 (27.5%)	NA
High fever (%)	24/51 (47.1%)	NA
Headache (%)	16/51 (31.4%)	NA
Pleurisy (%)	12/51 (23.5%)	NA
Peritonitis (%)	1/51 (2.0%)	NA
Pericarditis (%)	7/51 (13.7%)	NA
Arthritis (%)	35/51 (68.6%)	NA
Skin involvement (%)	32/51 (62.7%)	NA
Neuropsychiatric involvement (%)	14/51 (27.5%)	NA
Lung involvement (%)	5/51 (9.8%)	NA
Lupus nephritis (%)	25/55 (45.5%)	NA
Classification of biopsy-proven LN, number	Class II, 5; Class III, 1; Class IV, 6; Class V, 5; Class III + V, 1; Class IV + V, 3	NA
SELENA-SLEDAI score at disease onset *	12.0 (8.0–19.0, 47)	NA
Laboratory findings at disease onset		
WBC (x10^3/μl) *	4.2 (2.9–5.3, 43)	NA
Hemoglobin (g/dl) *	11.5 (10.0–12.2, 43)	NA
PLT (x10^4 /ul) *	18.3 (9.6–25.7, 43)	NA
CRP (mg/dl) *	0.2 (0.06–0.6, 43)	NA
Low C3 (%)	27/43 (62.8%)	NA
Low C4 (%)	22/43 (51.2%)	NA
Low CH50 (%)	26/43 (60.5%)	NA
Anti-dsDNA antibody positivity (%)	36/50 (72.0%)	NA
MEFV gene analysis		NA
The pattern of variants, number	E84K/–, 1; E84K/E148Q, 1; L110P/E148Q, 11; L110P/E148Q/E148Q, 2; L110P/L110P/E148Q/E148Q, 1; E148Q/–, 11; E148Q/E148Q, 1;	NA

	E148Q/R202Q, 1; E148Q/G304R, 1;<br>E148Q/P369S, 3; E148Q/S503C, 1;<br>R202Q/–, 1; G304R/–, 2;<br>P369S/R408Q,1; S503C/–, 1	
**Minor allele frequency for each *MEFV* variant**		
E84K (%)	2/110 (1.8%)	17/5194 (0.3%)
L110P (%)	15/110 (13.6%)	414/5192 (8.0%)
E148Q (%)	37/110 (33.6%)	1443/5160 (28.0%)
R202Q (%)	2/110 (1.8%)	196/5182 (3.8%)
G304R (%)	3/110 (2.7%)	118/5192 (2.3%)
P369S (%)	4/110 (3.6%)	380/5180 (7.3%)
R408Q (%)	1/110 (0.9%)	288/5194 (5.5%)
S503C (%)	2/110 (1.8%)	10/5198 (0.2%)

*Median (interquartile range, number) or number (percentages) are shown. P-values were established using Fisher's exact test or the Mann-Whitney U-test. # Genome database in East Asian population from gnomAD v3.1.2 (https://gnomad.broadinstitute.org). SLE: systemic lupus erythematosus; *MEFV*: *Mediterranean Fever*; SELENA-SLEDAI: Safety of Estrogens in Lupus Erythematosus National Assessment–Systemic Lupus Erythematosus Disease Activity Index; WBC: white blood cell count; PLT: Platelet; CRP: C-reactive protein.
